# ﻿*Goodyeramedogensis* (Orchidaceae), a new species from Tibet, China

**DOI:** 10.3897/phytokeys.189.77374

**Published:** 2022-02-14

**Authors:** Yi-Hua Tong1, Mei Sun*, Bing-Mou Wang, Huai-Zhen Tian

**Affiliations:** 1 Guangdong Provincial Key Laboratory of Applied Botany, South China Botanical Garden, Chinese Academy of Sciences, Guangdong, 510650, Guangzhou, China South China Botanical Garden, Chinese Academy of Sciences Guangzhou China; 2 Center of Conservation Biology, Core Botanical Gardens, Chinese Academy of Sciences, Guangzhou, 510650, China Core Botanical Gardens, Chinese Academy of Sciences Guangzhou China; 3 School of Life Sciences, East China Normal University, Shanghai, 200241, China East China Normal University Shanghai China; 4 Panyu Central Hospital, Guangzhou, 511402, China Panyu Central Hospital Guangzhou China

**Keywords:** Cranichideae, Jewel orchid, morphology, new species, Orchidoideae, phylogeny

## Abstract

A new species of *Goodyera* (Orchidaceae) from Tibet, China, *G.medogensis*, is described and illustrated here. Molecular phylogenetic results based on one nuclear (ITS) and two plastid markers (*matK* and *trnL-F*) support the recognition of *G.medogensis* as a new species in GoodyerasubsectionReticulum. Morphologically, the novelty is most similar to *G.biflora*, *G.vittata* and especially to *G.hemsleyana*, but differs by the thick grid lines of the reticulations with a diffused margin on the adaxial surface of the leaf blades, the inflorescence with more flowers, the obliquely obovate-rhombic petals, the yellow or yellowish labellum without a lamella on the blade, and the shorter trichomes on the floral bracts, sepals and ovary. Finally, a key to the species of Goodyerasubsect.Reticulum in China is also provided.

## ﻿Introduction

The genus *Goodyera* R. Br. (Orchidaceae) belongs to the subtribe Goodyerinae (Pridgeon et al. 2003; Chase et al. 2015), which includes three major subdivisions: the *Pachyplectron* clade, the *Goodyera* clade and the *Cheirosylis* clade (Chen et al. 2019). Different phylogenetic studies have revealed that *Goodyera* is polyphyletic (Juswara 2010; Hu et al. 2016; Chen et al. 2019), which has led Pace (2020) to subsequently propose a new generic arrangement for the *Goodyera* clade including 11 genera: *Goodyeras.s.*, *Cionisaccus* Breda, *Aspidogyne* Garay, *Microchilus* C. Presl, *Kreodanthus* Garay, *Lepidogyne* Blume, *Hylophila* Lindl., *Platylepis* A. Rich., *Eucosia* Blume, *Erythrodes* Blume, *Salacistis* Rchb. f. and *Paorchis* M. C. Pace. Since the available molecular phylogenetic results are based on few markers, and the quality and representativeness of samples are limited, in this study, we still accept a broad concept of *Goodyera*.

*Goodyeras.l.* consists of about 99 species distributed in Africa (Mozambique), Western Indian Ocean Islands, Asia, Southwest Pacific islands, Northeast Australia, Europe, Macaronesia (Madeira), North and Central America and the Caribbean (Chen et al. 2009; Zhou et al. 2020; Govaerts et al. 2021; Thiv et al. 2021). It is characterized by an elongate creeping rhizome, a cymbiform lip with a concave-saccate hypochile, sectile pollinia and one stigma.

There are 36 accepted species of *Goodyera* in China, of which 15 species are endemic (Chen et al. 2009; Guan et al. 2014; Jin and Yang 2015; Zhou et al. 2016; Liu et al. 2019; Wang et al. 2020; Zhou et al. 2020), and only *G.repens* (L.) R. Br. and *G.brachystegia* Hand.-Mazz. are distributed to the north of the Yangtze River. During a botanical survey in Medog County, Tibet Autonomous Region in January 2021, a few living plants with greenish reticulated leaves, resembling those of *Macodes* (Blume) Lindl., were collected and cultivated in Kunming Botanical Garden. Plants bloomed vigorously with many flowers in August. We also found flowering individuals in the field at the same time. The novelty is similar to *G.hemsleyana* King & Pantl. at first glance. However, it has greenish flowers with a yellow lip, while *G.hemsleyana* has pink flowers with a white lip. Besides, the reticulations on the leaf blades of the two species are also different.

Based on molecular phylogenetic evidence and a detailed examination of the morphological characters of our materials, the relevant types and a comprehensive array of additional herbarium specimens of similar species, we concluded that the plants discovered in Medog County in January 2021 represented a new species to science. It is described and illustrated here as *G.medogensis* H. Z. Tian, Y. H. Tong & B. M. Wang and included in a key to the species of Goodyerasubsect.Reticulum in China.

## ﻿Materials and methods

**Figure 1.**
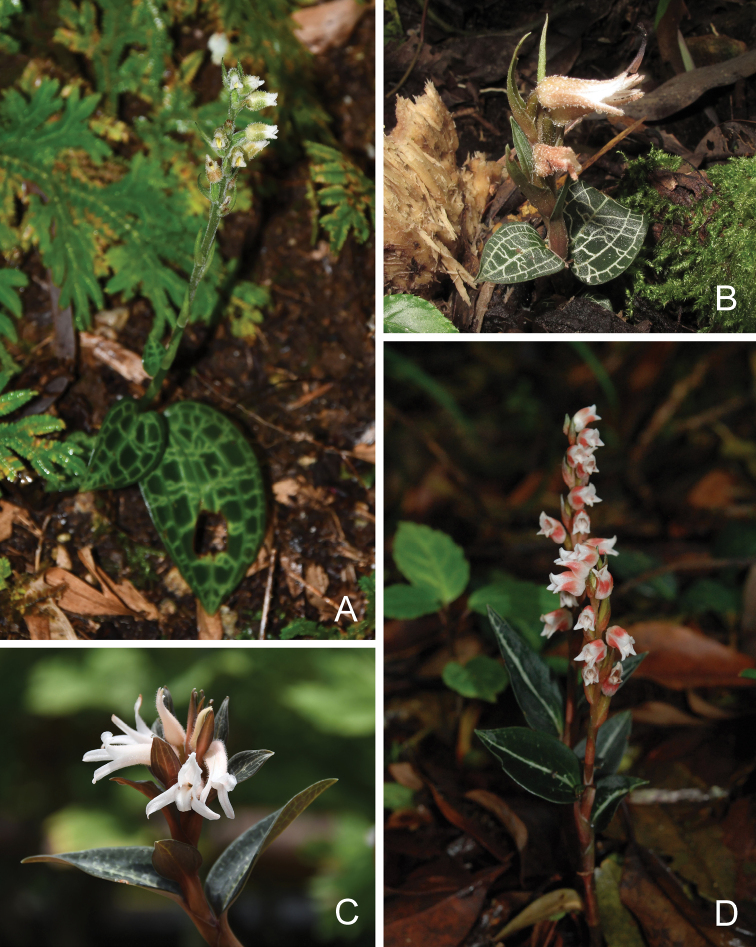
*Goodyeramedogensis* and three morphologically similar species *in situ***A***G.medogensis* (from Medog, Tibet) **B***G.hemsleyana* (from Malipo, Yunnan) **C***G.biflora* (from Medog, Tibet) **D***G.vittata* (from Yingjiang, Yunnan). Photographs **A, C, D** by Huai-Zhen Tian **B** by Chao Hu.

Based on our field observations of *Goodyera* in China in the past ten years, we identified three species that were closely related to the novelty, viz. *G.hemleyana*, *G.biflora* (Lindl.) Hook. f. and *G.vittata* Benth. ex Hook. f. (Fig. 1). Accordingly, we checked the types and additional material of these three taxa together with other pertinent specimens at HSNU, IBSC, K, KUN, LBG and PE. Photographs of specimens housed at AMES, AU, BR, CAL, CSFI, M and P were also examined. Among the target taxa, *G.biflora* is by far the most well-represented species in herbaria, and we did not find any additional specimens of the new species. Furthermore, we examined the distribution of multiple characters (e.g. leaf reticulation, flower number and indumentum, color and margin of the labellum) in all species of Goodyerasubsect.Reticulum in China, and these observations were summarized in the form of an identification key.

Voucher specimens of *G.medogensis* were collected in Medog County and preserved at the herbarium of South China Botanical Garden, Chinese Academy of Sciences (IBSC) and East China Normal University (HSNU). Fresh leaves used for molecular analyses were preserved in silica gel. The morphological description of the novelty is based on living material. Measurements were performed with a ruler (0.5 mm accuracy), and small plant parts were observed and measured under a stereo microscope (Mshot-MZ101).

To study the phylogenetic position of the new species within the genus *Goodyera*, three DNA fragments, viz. internal transcribed spacer (ITS) and two plastid DNA regions (*matK* and *trnL-F*), were selected for building the phylogenetic tree based on previous studies (Hu et al. 2016; Zhou et al. 2020). In total, 33 species represented by 80 samples of *Goodyera* were analyzed with one sample of *Zeuxineflava* (Wall. ex Lindl.) Benth. ex Hook. f. as the outgroup. All sequences were downloaded from GenBank except those of the new species. Species names and GenBank accession numbers are provided in the supplementary materials (Suppl. material 1: Table S1).

Total genomic DNA of the new species was extracted from silica gel-dried leaves using a modified CTAB method (Doyle and Doyle 1987). Polymerase Chain Reaction (PCR) amplification was carried out on TAKARA TP600 thermocycler (TAKARA BIO INC, Japan) using 25 μl reactions containing 12.5 μl 2× Taq PCR Master Mix (HuaGene, China), 8.5 μl ddH_2_O, 1.5 μl of each primer (10 μM) and 1 μl target DNA template. Detailed information of primers of relevant DNA fragments used in PCR amplification and sequencing, as well as the procedures of PCR, can be found in Suppl. material 1: Table S2. The resulting amplicons were visualized by horizontal agarose gel electrophoresis (1%), colored with GoldView I (Solarbio, China). Clearly distinguishable bands were recorded, and then the corresponding PCR products were sequenced by Shanghai HuaGene Biotech Co., Ltd (Shanghai, China).

Sequences were firstly assembled and edited with Seqman (DNA STAR package, Madison, WI, F USA) and then adjusted manually. Phylogenetic analysis was conducted using PhyloSuite ver. 1.2.2 (Zhang et al. 2020). Sequences were aligned with MAFFT (Katoh and Standley 2013). Ambiguously aligned fragments were removed using Gblocks (Talavera and Castresana 2007) with all parameters at their default settings. Next, *matK* and *trnL-F* were concatenated as well as ITS, *matK* and *trnL-F* respectively to two datasets. Thus, three datasets were constructed in total: the cpDNA dataset (*matK* and t*rnL-F*), the nrDNA dataset (ITS) and the nr+cpDNA dataset (ITS, *matK* and *trnL-F*). The three datasets were analyzed by using Bayesian inference (BI) and maximum likelihood (ML) methods respectively. Best-fit evolutionary models for Mrbayes and IQ-TREE were selected under the Bayesian Information Criterion (BIC) using ModelFinder (Kalyaanamoorthy et al. 2017). The best-fit models for the Maximum likelihood (ML) analysis are K80+R2 (ITS) and K3Pu+F+R2 (cpDNA, nr+cpDNA), and for Bayesian inference (BI) they are K2P+G4 (ITS) and GTR+F+G4 (cpDNA, nr+cpDNA).

Based on these models, the Maximum Likelihood (ML) analysis was performed with IQ-TREE (Nguyen et al. 2015) for 10000 ultrafast (Minh et al. 2013) bootstraps, and Bayesian Inference (BI) phylogenies were inferred using MrBayes 3.2.6 (Ronquist et al. 2012). BI analysis consisted of two simultaneous runs and four simultaneous Markov Chain Monte Carlo (MCMC) chains, and ran for 3000000 generations with chain sampling every 1000 generations. The average deviation of split frequencies fell below 0.01, and initial 25% of sampled data were discarded as burn-in. The phylogenetic trees were visualized and modified in FigTree version 1.4.3 (Rambaut 2016).

## ﻿Results

**Figure 2.**
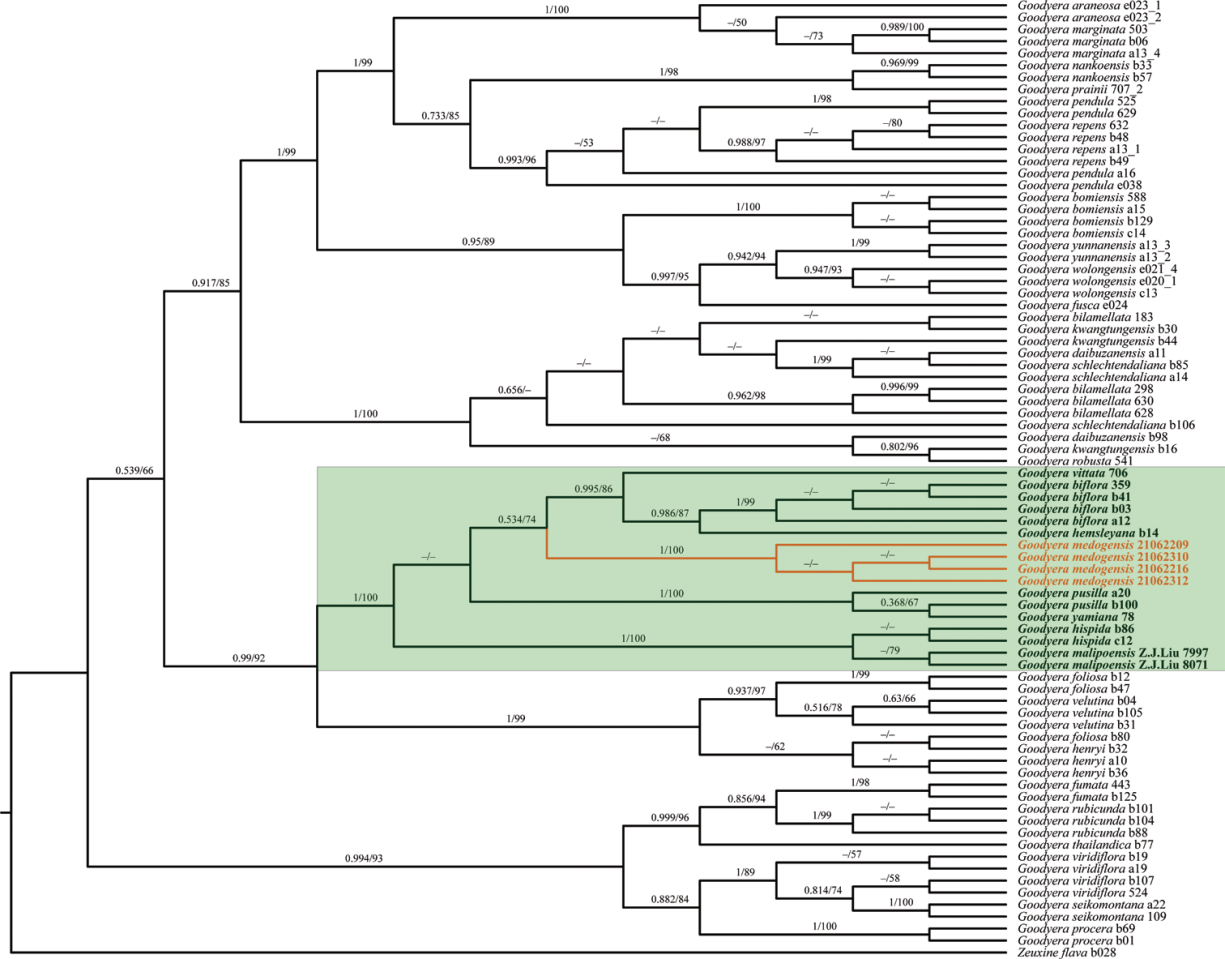
Phylogenetic tree of *Goodyera* species inferred by Bayesian and maximum likelihood analyses based on the nrDNA (ITS) dataset. Posterior probability (PP) ≥ 0.50 in BI analysis and bootstrap (BS) % values ≥ 50 in ML analysis are indicated above the branches. Dashes mean the nodes are not supported, i.e. the BS value < 50% in the ML analysis or PP < 0.50 in the BI analysis. The samples of the new species are highlighted in orange, and other species of sect. Reticulum are highlighted in bold. The clade of Goodyerasect.Reticulum is highlighted with the green rectangle.

**Figure 3.**
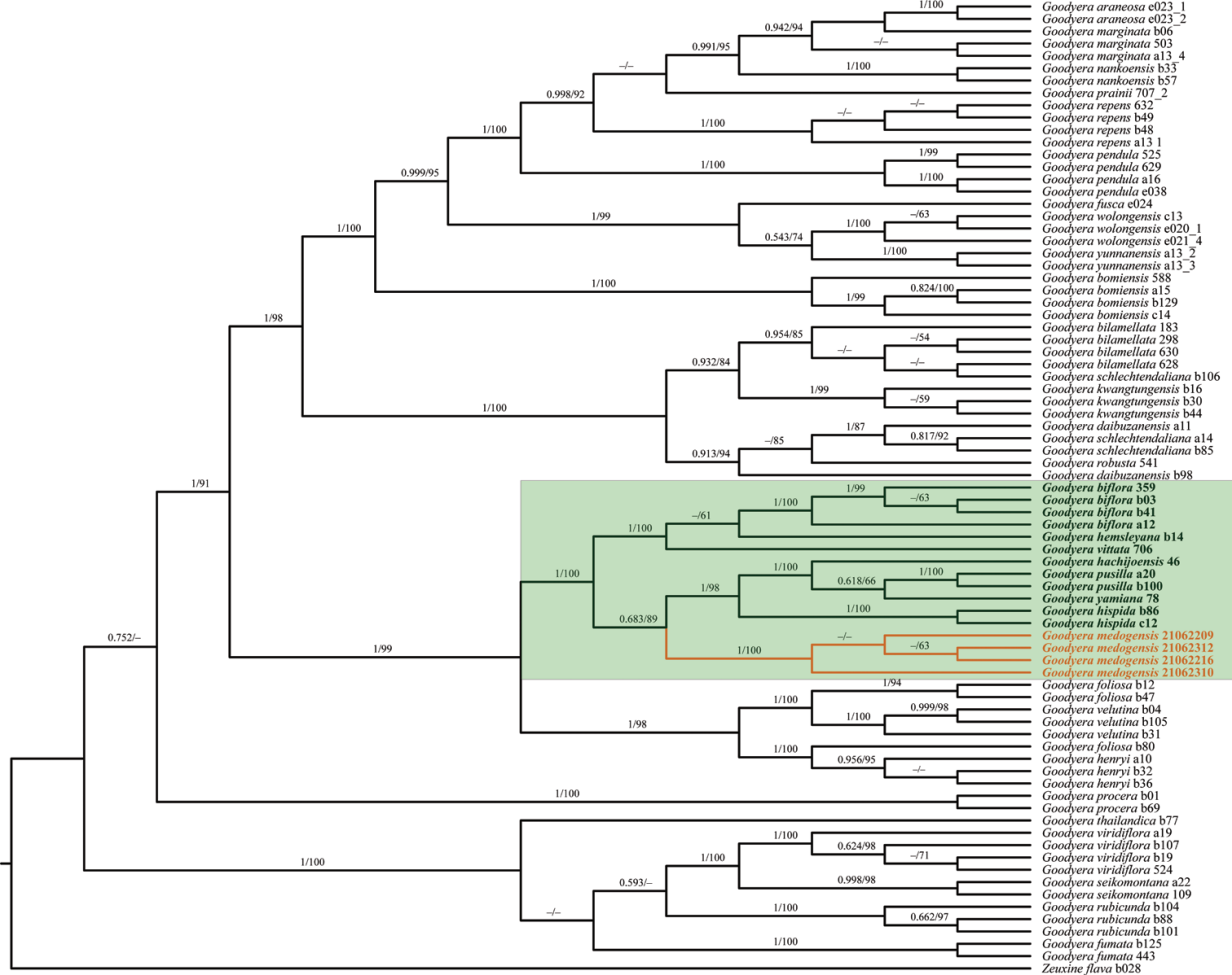
Phylogenetic tree of *Goodyera* species inferred by Bayesian and maximum likelihood analyses based on the cpDNA (*matK* + *trnL-F*) datasets. Posterior probability (PP) ≥ 0.50 in BI analysis and bootstrap (BS) % values ≥ 50 in ML analysis are indicated above the branches respectively. Dashes mean the nodes are not supported, i.e. the BS value < 50% in the ML analysis or PP < 0.50 in the BI analysis. The samples of the new species are highlighted in orange, and other species of sect. Reticulum are highlighted in bold. The clade of Goodyerasect.Reticulum is highlighted with the green rectangle.

**Figure 4.**
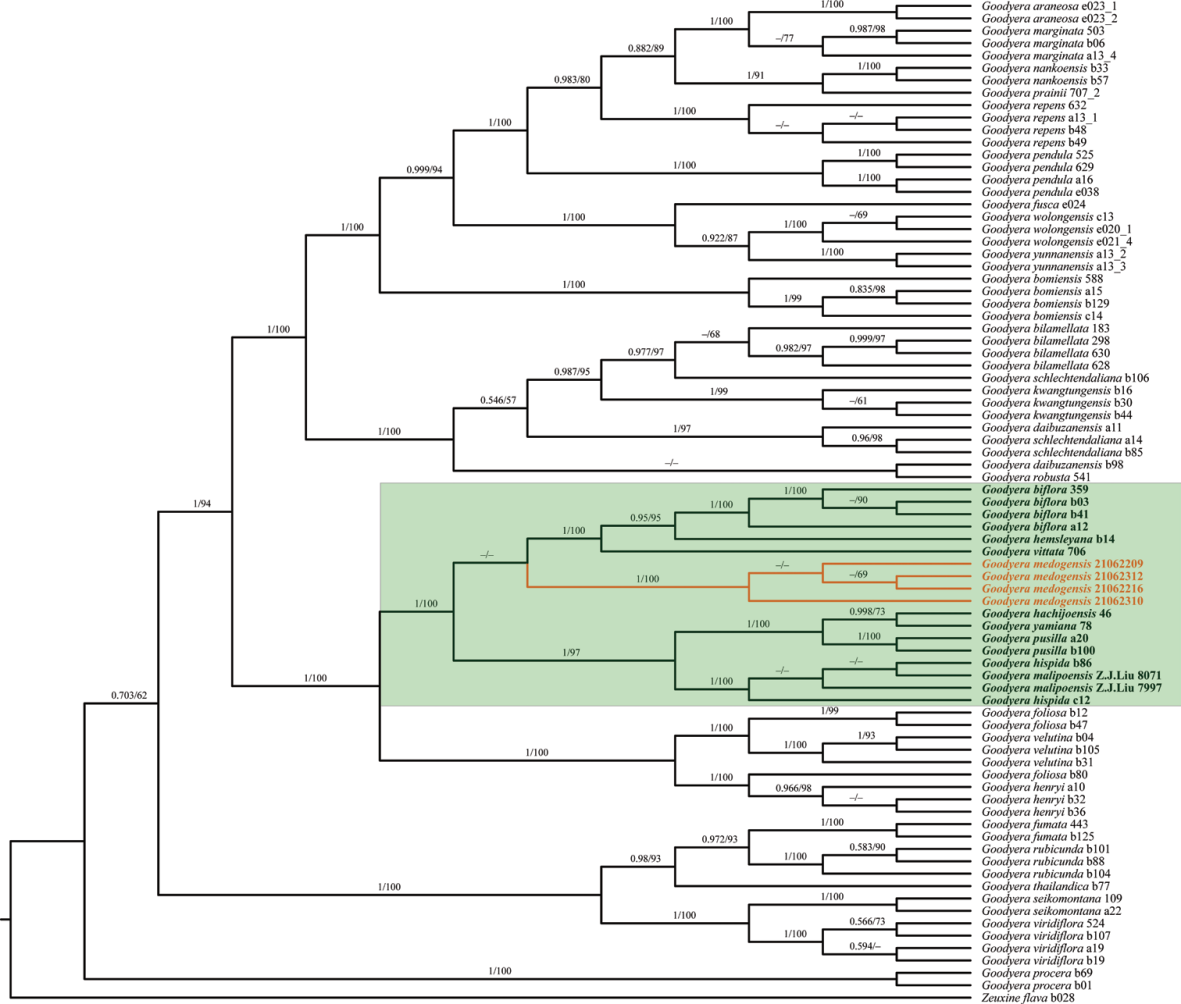
Phylogenetic tree of *Goodyera* species inferred by Bayesian and maximum likelihood analyses based on the nr + cpDNA (including ITS, *matK* and *trnL-F*) datasets. Posterior probability (PP) ≥ 0.50 in BI analysis and bootstrap (BS) % values ≥ 50 in ML analysis are indicated above the branches respectively. Dashes mean the nodes are not supported, i.e. the BS value < 50% in the ML analysis or PP < 0.50 in the BI analysis. The samples of the new species are highlighted in red, and other species of sect. Reticulum are highlighted in bold. The clade of Goodyerasect.Reticulum is highlighted with the green rectangle.

Our ML and BI phylogenetic trees constructed from the three datasets showed that our four samples of *G.medogensis* cluster into one separate subclade (Figs 2–4) which is nested in the clade of GoodyerasubsectionReticulum S. W. Chung & C. H. Ou (Hu et al. 2016) consisting of eight other species, viz. *G.biflora*, *G.hachijoensis* Yatabe, *G.hemsleyana*, *G.hispida* Lindl., *G.malipoensis* Q. X. Guan & S. P. Chen, *G.pusilla* Bl., *G.vittata* and *G.yamiana* Fukuy.. Thus, the results of phylogenetic analyses support the recognition of *G.medogensis* as a new species belonging to the subsection Reticulum, and has close relationship with *G.biflora*, *G.hemsleyana* and *G.vittata* (Fig. 1).

### ﻿Taxonomic treatment

#### ﻿ Goodyera medogensis

Taxon classificationPlantaeAsparagalesOrchidaceae

H. Z. Tian, Y. H. Tong & B. M. Wang sp. nov.

1D1645D3-6A8A-5344-BC81-BC3F58A5B373

urn:lsid:ipni.org:names:77254838-1

Figs 5 , 6

##### Type.

China. Tibet Autonomous Region: Medog County, Renqingbeng Temple, under evergreen broad-leaved forest, cultivated at Kunming Botanical Garden, 3 August 2021 (ﬂ.), *B. M. Wang TYH-2523* (holotype: IBSC, isotype: HSNU).

##### Diagnosis.

Similar to *G.hemsleyana*, but distinguished by the greenish thick reticulations on the adaxial surface of leaf blades with diffused margin (vs. white thin reticulations with clear margin), inflorescence with more flowers ((6–)12–15 vs. 4–10), petals obliquely obovate-rhombic (vs. obliquely ovate-oblong), labellum yellow or yellowish (vs. white, with light greenish to pinkish tinge at apex) without lamella on blade (vs. with a low bi-lamellate callus), and floral bracts, sepals as well as ovary with shorter trichomes.

**Figure 5.**
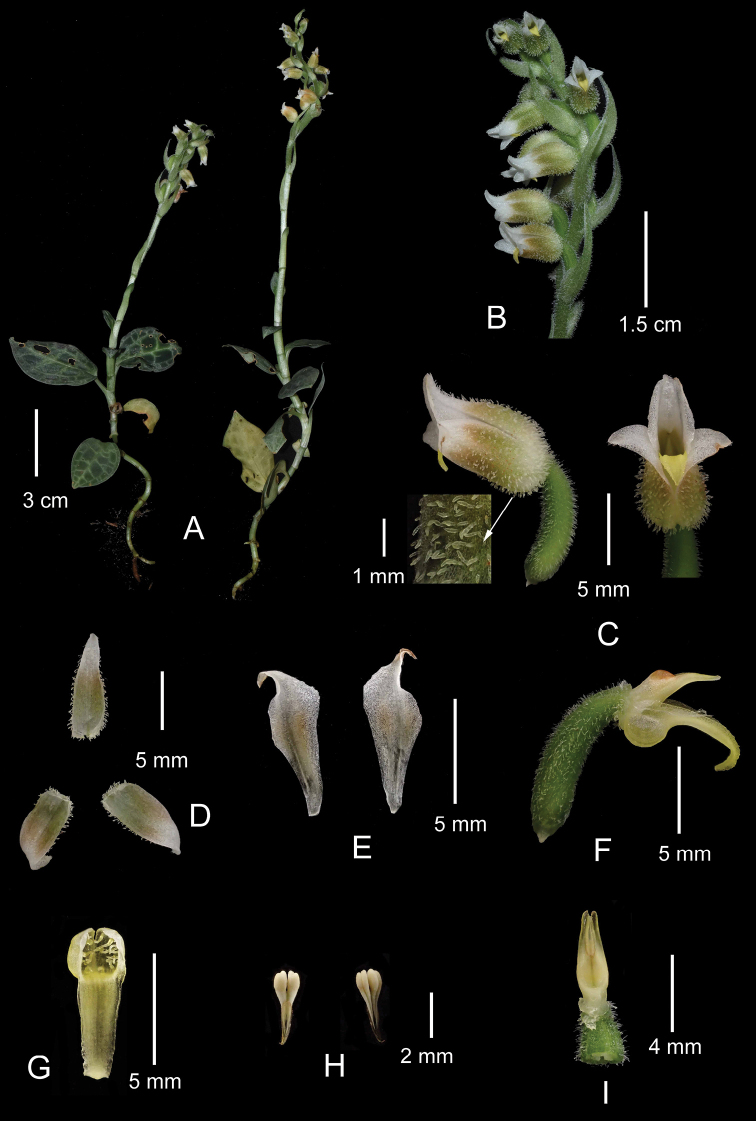
*Goodyeramedogensis***A** habit **B** inflorescence **C** flower, lateral (left) and front (right) view, with the arrow showing close-up of trichomes on abaxial surface of sepals **D** sepals **E** petals **F** column with labellum, anther and ovary **G** labellum **H** pollinarium **I** column with part of the ovary. Photographs by Yi-Hua Tong.

##### Description.

Terrestrial herb, 12–25 cm tall. Rhizome 4–6 cm long, 1.5–3 mm in diam., greenish, rooting at nodes. Roots fleshy, 0.7–7 cm long, yellowish brown, with minute root hairs. Stem erect, terete, 4–9 cm long, 2.7–4 mm in diam., pale green, glabrous, with few sheaths at base formed by withered bases of petioles. Leaves 3–7; petiole 1.1–1.6 cm long, sheathing at base; lamina ovate, 1.9–4.2 × 1.5–2.6 cm, obtuse at base, acute at apex, adaxially green to bluish green with greenish-white reticulations, grid lines thick, margin diffused, transverse ones 5–7, abaxially pale green, 5–7-veined. Inflorescence a terminal raceme, laxly (6–)12–15-flowered, spirally arranged, pubescent; peduncle 4–6.5 cm long, pubescent, with 2–3 sheathing bracts; sheathing bracts 1.5–1.9 × 0.6–0.8 cm, oblong-lanceolate, acute at apex, pale green, clasping, more or less pubescent, especially on the margin, 3–5-veined; rachis 3.5–7 cm long, pubescent. Floral bracts ovate-lanceolate, 1.4–1.7 × 0.5–0.6 cm, acuminate to acute at apex, pale green, longer than ovary, sometimes shortly ciliate at margins, pubescent abaxially, trichomes multicellular, up to 0.8 mm long, glabrous adaxially, 3-veined. Flowers resupinate, opening weakly, 8–10 mm long. Sepals 1-veined, acute at apex, olive greenish, with reddish or brownish tinge when old, with dense clavate trichomes outside, trichomes up to 0.5 mm long; dorsal sepal ovate-lanceolate, 8–9 × 2–3 mm, forming a hood with the petals; lateral sepals ovate-lanceolate, 8–9 × 3.5–4 mm. Petals obliquely obovate-rhombic, 8–8.5 × 3–3.5 mm, acuminate to acute at apex, white, with reddish or brownish tinge at central part, glabrous, 1-veined. Labellum oblong-ovate, 6–7 mm long, yellow or yellowish; hypochile with a sac ca. 1.5–2.5 × 2.5–3.5 mm, with glandular hairs inside; epichile oblong to oblong-lanceolate, entire, 5–6.5 × 2–3 mm, margins slightly undulate, obtuse to subacute at apex. Column cylindrical, ca. 1 mm long; rostellum 3.5–4 mm long, bifid, acuminate at apex. Stigma suborbicular, ca. 1 × 1 mm, entire. Anther yellowish brown, ovate, ca. 2.5 × 1.5 mm. Pollinarium 3–3.5 mm long; pollinia 2, oblong-obclavate, 0.8–1 mm long, dull yellowish white, sectile, bifid; caudicles 1.5–1.7 mm long; viscidium narrowly ovate-oblong, 2–3× ca. 0.5 mm, acute at apex, membranous. Ovary plus pedicel terete, 6–7.5× ca. 2 mm, pale green, twisted, pubescent, trichomes blunt, multicellular, consisting of 5–7 cells. Fruit not seen.

**Figure 6.**
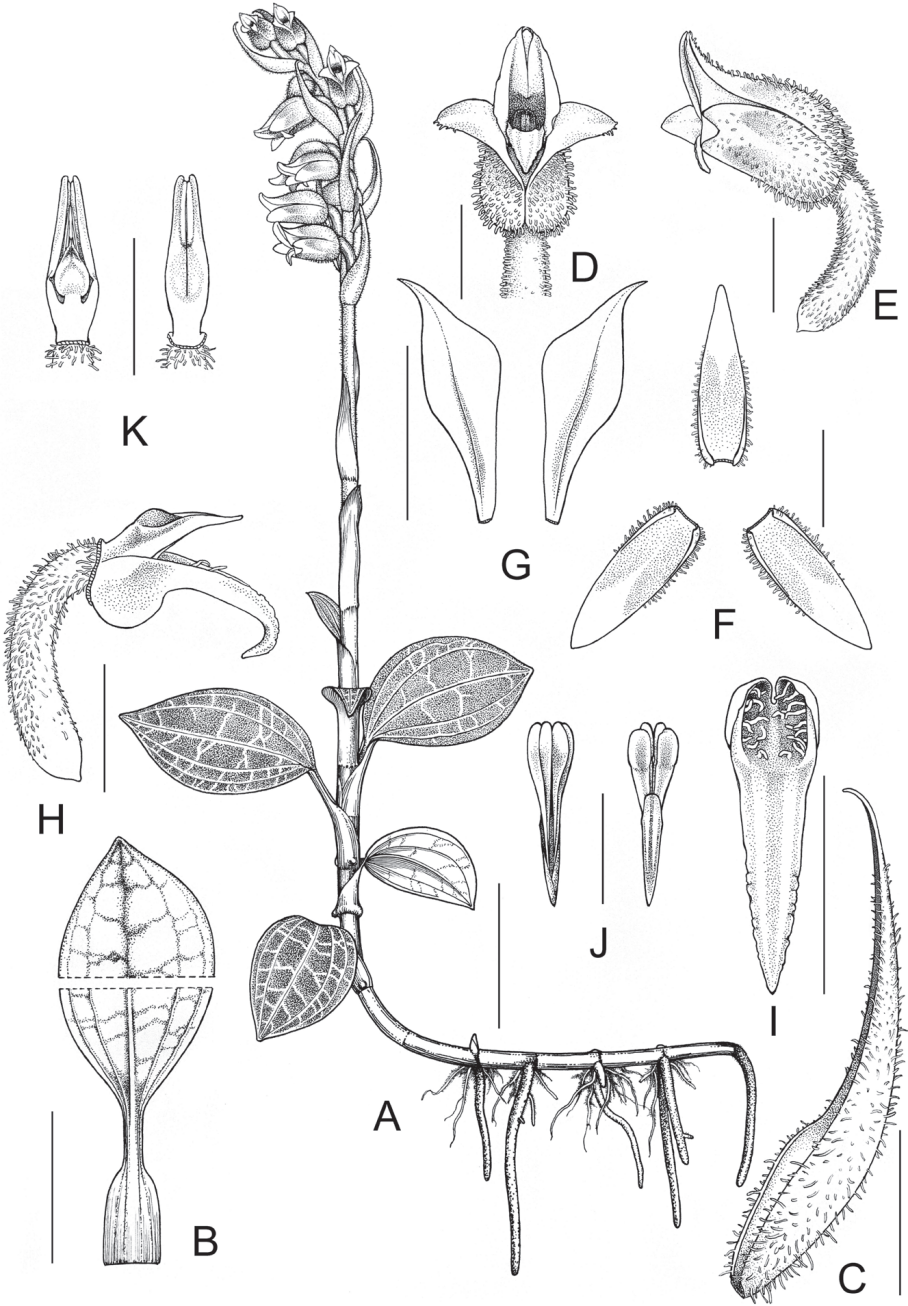
*Goodyeramedogensis***A** habit **B** leaf **C** bract **D** flower, front view **E** flower, lateral view **F** sepals **G** petals **H** column with labellum, anther and ovary **I** labellum **J** pollinarium **K** column with part of the ovary. Scale bars: 3 cm (**A**); 2 cm (**B**); 5 mm (**C–I**); 4 mm (**K**); 2 mm (**J**). Drawn by Jun Cai.

##### Etymology.

The species epithet refers to the type locality, Medog County.

##### Vernacular name.

墨脱斑叶兰 (Chinese pinyin: mò tuō bān yè lán).

##### Distribution and habitat.

This species is currently known only from Medog County, Tibet, China. It grows under evergreen broad-leaved forests at elevations of 1600–2300 m.

##### Conservation status.

During our three surveys in January, June and August 2021, *Goodyeramedogensis* was found in the forests of Medog Town and Beibeng Township of Medog County, where we counted a minimum of 200 individuals. However, since the population assessment of this species in the whole Medog County has not been made, conservation status of this new species is best classified as ‘Data Deficient’ (DD) (IUCN Standards and Petitions Committee 2019). It occurs within a conservation area, and no threats from logging, tourism or poaching have been recorded until now.

##### Phenology.

Flowering in July-August.

##### Additional specimens examined.

*Goodyeramedogensis* (paratypes): China: Tibet Autonomous Region, Medog County, Bari village, elev. 1750 m, 23 June 2021, cultivated in East China Normal University, 10 August 2021 (ﬂ.), *J. Huang & M. Sun 21062310* (HSNU); ibid., Medog village, 19 August 2021, *H. Z. Tian et al. 21081914* (HSNU); ibid., Gelin village, 21 August 2021, *H. Z. Tian et al. 21082102* (HSNU).

*Goodyerahemsleyana*: India: Senchal, 2100 m, July 1892, *Pantling 215* (lectoype: CAL0000000625 (photo); isolectotypes: CAL0000000624 (photo), K00387611, BR0000006573508 (photo), M0226196 (photo), AMES00090573 (photo), P00333538 (photo)).

*Goodyerabiflora*: Nepal: sin. loc., *Wallich*, *Cat. no. 7379* (holotype: K000364600; isotype: K001127259). India: sin. loc., 1900, *J. F. Duthie 24164* (K); Mussorie, July 1901, *P. W. Mackinnon 25408* (AMES02091486, photo); Himachal Pradesh, Shimla, 1524 m, 30 June 1886, *H. Collett. 325* (K); Himalaya, 1981 m, 1844, *M. P. Edgeworth 58* (K). China: Tibet, Gyirong Valley, Tsangpo Valley, 2743 m, 6 August 1935, *F. Kingdom-Ward 12159* (K); Tibet, Bome, 2 July 1952, *P. C. Tsoong 6699* (PE00339319, PE00339320); Anhui, Jinzhai, 1600 m, 6 August 1986, *Plant Resource Team D0062* (PE); Guangdong, Fengkai, 16 June 1974, *Yue Seven Four 5295* (IBSC0627265); Guangdong, Xinyi, 15 April 1931, *S. P. Ko 51307* (IBSC0627264, IBSC0627266, IBSC0627267, PE00339323, PE00339324); Guangdong, Ruyuan, 27 November 1957, *L. Teng 5866* (IBSC0627260); Guizhou, Fanjing Mt., 10 September 1987, *China-USA Scientific Research Team s.n.* (PE00339322); Guizhou, Rongjiang, 10 July 1974, *Anonymous 74-913* (IBSC0627261); Henan, Xin County, 10 August 2013, *C. S. Zhu*, *S. J. Li*, *X. L. Hou*, *S. X. Zhu*, *J. M. Li 130808106* (AU066898, photo); Henan, Shangcheng, 19 June 1984, *Plant Resource Research Team D0546* (PE00850731); Hubei, Huanggang, 2 October 2018, *X. X. Zhu*, *L. L. Shi*, *S. S. Duan*, *M. J. Hu*, *Q. Lü ZXX18494* (KUN1444572, photo); Hubei, Shiyan, 8 July 2013, *S. L. Li GanQL486* (KUN1458343, photo); Hunan, Chengbu, 22 May 2020, *L. Wu*, *W. J. Liu*, *C. F. Deng 10403* (CSFI071975, photo); Hunan, Sangzhi, 600 m, 22 June 1991, *Q. Lin 714* (IBSC0627263); Hunan, Sangzhi, 11 June 2019, *X. Li*, *C. F. Deng*, *J. L. Li 190611132* (CSFI072175, photo); Hunan, Cili, 2 October 1984, *G. X. Xing & Q. Xia 05571* (PE00339318); Sichuan, Hechuan to Mabian, 1934, *S. L. Sun 5590* (PE00339322); Sichuan, s. d., *P. C. Tsoong 3981* (PE01849749); Yunnan, Wenshan, 25 September 1958, *H. T. Tsai 58-8126* (KUN0022202); Zhejiang, West Tianmu, 2 July 1925, *D. X. Zhang* 266 (LBG00108146).

*Goodyeravittata*: India: Sikkim Himalaya, *J. D. Hooker 336* (holotype: K000364605); Singalelah Range, 2438 m, July 1896, *R. Pantling 410* (AMES02091540, photo). China: Tibet, Zayu, 25 July 1980, 2100 m, *Z. C. Ni*, *Y. Z. Wang*, *D. Ci et al. 0757B* (PE00339514); Yunnan, Yingjiang, 14 August 2012, *H. Z. Tian & C. Hu 706* (HSNU).

### ﻿Key to species of Goodyerasubsect.Reticulum in China

**Table:** 

1	Inflorescence with more than 20 flowers; dorsal sepal less than 6 mm long	**2**
–	Inflorescence with less than 20 flowers; dorsal sepal more than 6 mm long	**6**
2	Leaves green, without white or pale green venation on adaxial surface	***G.yamiana***
–	Leaves with white or pale green venation on adaxial surface	**3**
3	Margins of epichile and petals irregularly denticulate	***G.pusilla***
–	Margins of labellum and petals entire	**4**
4	Flowers glabrous	***G.hachijoensis***
–	Flowers pubescent	**5**
5	Flower diameter 6–7 mm	***G.malipoensis***
–	Flower diameter 3–4 mm	***G.hispida***
6	Inflorescence mostly with 2 flowers, sometimes up to 6; dorsal sepal 20–25 mm long	***G.biflora***
–	Inflorescence mostly with 4–15 flowers; dorsal sepal 3–14 mm long	**7**
7	Leaves adaxially with a white band along midvein	***G.vittata***
–	Leaves adaxially with white or greenish reticulate venation	**8**
8	Leaves adaxially with thin and white reticulations with clear margins; labellum white, with light greenish to pinkish tinge at apex	***G.hemsleyana***
–	Leaves adaxially with thick and greenish reticulations with diffused margins; labellum yellow or yellowish	***G.medogensis***

## ﻿Discussion

The new species has the typical features of Goodyerasect.Reticulum, i.e., the leaves have reticulations on adaxial surface, and lateral sepals are not reflexed backwards. According to Hu et al. (2016), sect. Reticulum is one of the four sections of *Goodyera*, and can be further divided into two subsections, viz., G.subsect.Reticulum S. W. Chung & C. H. Ou and G.subsect.Foliosum S. W. Chung & C. H. Ou. Based on morphology, *G.medogensis* is probably most closely related to *G.hemsleyana*. However, considering the conflicting results shown by nrDNA and cpDNA, the position of the new species within subsect. Reticulum remains elusive.

## Supplementary Material

XML Treatment for Goodyera medogensis

## Supplementary materials

Supplementary material 1 Table S1, S2. Supplementary file of *Goodyeramedogensis* (Orchidaceae), a new species from Tibet, China Data type phylogenetic Explanation note Details of materials for phylogenetic study and primers for sequence amplication and sequencing. https://binary.pensoft.net/file/644910This dataset is made available under the Open Database License (http://opendatacommons.org/licenses/odbl/1.0/). The Open Database License (ODbL) is a license agreement intended to allow users to freely share, modify, and use this Dataset while maintaining this same freedom for others, provided that the original source and author(s) are credited.Yi-Hua Tong, Mei Sun, Bing-Mou Wang, Huai-Zhen Tian

## References

[B1] Chase MW Cameron KM Freudenstein JV Pridgeon AM Salazar G Van den Berg C Schuiteman A (2015) An updated classification of Orchidaceae. Botanical Journal of the Linnean Society 177(2): 151–174. 10.1111/boj.12234

[B2] Chen SC Lang KY Gale SW Cribb PJ Ormerod P (2009) *Goodyera* R. Br. In: Wu ZY Raven PH Hong DY (Eds) Flora of China (Vol.25). Science Press, Beijing & Missouri Botanical Garden Press, St. Louis, 45–54.

[B3] Chen SP Tian HZ Guan QX Zhai JW Zhang GQ Chen LJ Liu ZJ Lan SR Li MH (2019) Molecular systematics of Goodyerinae (Cranichideae, Orchidoideae, Orchidaceae) based on multiple nuclear and plastid regions. Molecular Phylogenetics and Evolution 139: e106542. 10.1016/j.ympev.2019.106542 31229601

[B4] Doyle JF Doyle JL (1987) A rapid DNA isolation procedure for small quantities of fresh leaf tissue. Phytochemical Bulletin 19(1): 11–15.

[B5] Govaerts R Bernet P Kratochvil K Gerlach G Carr G Alrich P Pridgeon AM Pfahl J Campacci MA Holland Baptista D Tigges H Shaw J Cribb P George A Kreuz K Wood J (2021) World Checklist of Orchidaceae. Facilitated by the Royal Botanic Gardens, Kew csience. http://wcsp.science.kew.org/ [Accessed on 09.01.2022]

[B6] Guan QX Chen GZ Li MH Chen SP (2014) *Goodyeramalipoensis* (Cranichideae, Orchidaceae), a new species from China: Evidence from morphological and molecular analyses. Phytotaxa 186(1): 51–60. 10.11646/phytotaxa.186.1.4

[B7] Hu C Tian HZ Li HQ Hu AQ Xing FW Bhattacharjee A Hsu TC Kumar P Chung SW (2016) Phylogenetic analysis of a ‘jewel orchid’ genus *Goodyera* (Orchidaceae) based on DNA sequence data from nuclear and plastid regions. PLoS ONE 11(2): e0150366. 10.1371/journal.pone.0150366 PMC4771202 26927946

[B8] IUCN Standards and Petitions Committee (2019) Guidelines for Using the IUCN Red List Categories and Criteria. Version 14. Prepared by the Standards and Petitions Committee, 116 pp. http://www.iucnredlist.org/documents/RedListGuidelines.pdf

[B9] Jin XH Yang Y (2015) Species catalogue of China (Vol.1) Plants. Spermatophytes (1). Science Press, Beijing, 242–245. [in Chinese]

[B10] Juswara LS (2010) Phylogenetic analyses of subtribe Goodyerinae and revision of GoodyerasectionGoodyera (Orchidaceae) from Indonesia, and fungal association of GoodyerasectionGoodyera. Ph. D. Thesis, The Ohio State University, 486 pp.

[B11] Kalyaanamoorthy S Minh BQ Wong TKF von Haeseler A Jermiin LS (2017) ModelFinder: Fast model selection for accurate phylogenetic estimates. Nature Methods 14(6): 587–589. 10.1038/nmeth.4285 28481363 PMC5453245

[B12] Katoh K Standley DM (2013) MAFFT multiple sequence alignment software version 7: Improvements in performance and usability. Molecular Biology and Evolution 30(4): 772–780. 10.1093/molbev/mst010 23329690 PMC3603318

[B13] Liu YW Zhou XX Schuiteman A Kumar P Hermans J Chung SW Tian HZ (2019) Taxonomic notes on *Goodyera* (Goodyerinae, Cranichideae, Orchidoideae, Orchidaceae) in China and an addition to orchid flora of Vietnam. Phytotaxa 395(1): 027–034. 10.11646/phytotaxa.395.1.3

[B14] Minh BQ Nguyen MA von Haeseler A (2013) Ultrafast approximation for phylogenetic bootstrap. Molecular Biology and Evolution 30(5): 1188–1195. 10.1093/molbev/mst024 23418397 PMC3670741

[B15] Nguyen LT Schmidt HA von Haeseler A Minh BQ (2015) IQ-TREE: A fast and effective stochastic algorithm for estimating maximum-likelihood phylogenies. Molecular Biology and Evolution 32(1): 268–274. 10.1093/molbev/msu300 25371430 PMC4271533

[B16] Pace MC (2020) A recircumscription of *Goodyera* (Orchidaceae), including the description of *Paorchis* gen. nov., and resurresction of *Cionisaccus*, *Eucosia*, and *Salacistis*. Brittonia 72(3): 257–267. 10.1007/s12228-020-09623-y

[B17] Pridgeon AM Cribb PJ Chase MW Rasmussen FR (2003) Genera Orchidacearum, Vol. 3: Orchidoideae (Part 2). Vanilloideae. Oxford University Press, Oxford, 94–98.

[B18] Rambaut A (2016) FigTree 1.4.3. Computer program distributed by the author. http://tree.bio.ed.ac.uk/software/figtree

[B19] Ronquist F Teslenko M van der Mark P Ayres DL Darling A Höhna S Larget B Liu L Suchard MA Huelsenbeck JP (2012) MrBayes 3.2: Efficient Bayesian phylogenetic inference and model choice across a large model space. Systematic Biology 61(3): 539–542. 10.1093/sysbio/sys029 22357727 PMC3329765

[B20] Talavera G Castresana J (2007) Improvement of phylogenies after removing divergent and ambiguously aligned blocks from protein sequence alignments. Systematic Biology 56(4): 564–577. 10.1080/10635150701472164 17654362

[B21] Thiv M Gouveia M Menezes de Sequeira M (2021) The Madeiran laurel forest endemic *Goodyeramacrophylla* (Orchidaceae) is related to American orchids. Anales del Jardín Botánico de Madrid 78(2): e116. 10.3989/ajbm.2605

[B22] Wang XL Chen J Jin XH (2020) *Goodyerananshanensis* (Orchidaceae, Orchidoideae, Cranichideae, Goodyerinae), a new species from Hunan, China. Phytotaxa 460(4): 296–300. 10.11646/phytotaxa.460.4.7

[B23] Zhang D Gao F Jakovlić I Zou H Zhang J Li WX Wang GT (2020) PhyloSuite: An integrated and scalable desktop platform for streamlined molecular sequence data management and evolutionary phylogenetics studies. Molecular Ecology Resources 20(1): 348–355. 10.1111/1755-0998.13096 31599058

[B24] Zhou XX Cheng ZQ Liu QX Zhang JL Hu AQ Huang MZ Hu C Tian HZ (2016) An updated checklist of Orchidaceae for China, with two new national records. Phytotaxa 276(1): 1–148. 10.11646/phytotaxa.276.1.1

[B25] Zhou S Zhou XX Jin Y Schuiteman A. Kumar P Yuan D Tian HZ (2020) *Goodyeraaraneosa*: a new species of Orchidaceae from Sichuan, China and its affinities. Plant Biosystems-An International Journal Dealing with all Aspects of Plant Biology 155(2): 344–349. 10.1080/11263504.2020.1747563

