# Supplementary material for: ﻿Goodyeramedogensis (Orchidaceae), a new species from Tibet, China
Source: PhytoKeys. 2022 Feb 14;189:141–54. doi: 10.3897/phytokeys.189.77374 (PMC8860984; doi:10.3897/phytokeys.189.77374)
Supplement: Supplementary material 1 — Table S1, S2. Supplementary file of Goodyeramedogensis (Orchidaceae), a new species from Tibet, China [file phytokeys-189-141-s001.docx]

Table S1. Voucher specimens used in phylogenetic analysis.

| Taxon | Voucher | Collector | Locality | nrITS | trnL-F | matK |
| --- | --- | --- | --- | --- | --- | --- |
| *Goodyera araneosa* | e023-1 (HSNU) | Tian, Zhang & Zhou | Wanglang, Sichuan, China | MN472742 | MN544775 | MN563606 |
| *Goodyera araneosa* | e023-2 (HSNU) | Tian, Zhang & Zhou | Wanglang, Sichuan, China | MN472743 | MN544776 | MN563607 |
| *Goodyera biflora* | 359 (HSNU) | Chung | Hsinchu, Taiwan, China | KT343986 | KT385501 | KT385588 |
| *Goodyera biflora* | a12 (HSNU) | Tian | Tibet, China | KT343987 | KT385498 | KT385585 |
| *Goodyera biflora* | b03 (HSNU) | Hu | Linan, Zhejiang, China | KT343988 | KT385499 | KT385586 |
| *Goodyera biflora* | b41 (HSNU) | Hu & Li | Sangzhi, Hunan, China | KT343989 | KT385500 | KT385587 |
| *Goodyera bilamellata* | 183 (HSNU) | Chung | Hualien, Taiwan, China | KT343990 | KT385502 | KT385589 |
| *Goodyera bilamellata* | 298 (HSNU) | Chung | Taipei, Taiwan, China | KT343991 | KT385503 | KT385590 |
| *Goodyera bilamellata* | 628 (HSNU) | Chung | Hsinchu, Taiwan, China | KT343992 | KT385504 | KT385591 |
| *Goodyera bilamellata* | 630 (HSNU) | Chung | Nantou, Taiwan, China | KT343993 | KT385505 | KT385592 |
| *Goodyera bomiensis* | 588 (HSNU) | Chung | Hsinchu, Taiwan, China | KT343994 | KT385506 | KT385593 |
| *Goodyera bomiensis* | a15 (HSNU) | Tian | Nyingchi, Tibet, China | KT343995 | KT385507 | KT385594 |
| *Goodyera bomiensis* | b129 (HSNU) | Hu & Li | Huoshan, Anhui, China | KT343996 | KT385508 | KT385595 |
| *Goodyera bomiensis* | c14 (HSNU) | Tang | Xichang, Sichuan, China | KT343997 | KT385509 | KT385596 |
| *Goodyera daibuzanensis* | a11 (HSNU) | Tian | Zayu, Tibet, China | KT343999 | KT385510 | KT385597 |
| *Goodyera daibuzanensis* | b98 (HSNU) | Tian | Pingtung, Taiwan, China | KT344000 | KT385511 | KT385598 |
| *Goodyera foliosa* | b12 (HSNU) | Hu | Malipo, Yunnan, China | KT344004 | KT385513 | KT385600 |
| *Goodyera foliosa* | b47 (HSNU) | Hu | Guilin, Guangxi, China | KT344005 | KT385514 | KT385601 |
| *Goodyera foliosa* | b80 (HSNU) | Ye | Simao, Yunnan, China | KT344007 | KT385516 | KT385603 |
| *Goodyera fumata* | 443 (HSNU) | Chung | Taipei, Taiwan, China | KT344010 | KT385519 | KT385606 |
| *Goodyera fumata* | b125 (HSNU) | Tian & Dong | Wuzhishan, Hainan, China | KT344011 | KT385520 | KT385607 |
| *Goodyera fusca* | e024 (HSNU) | Jin | Yadong, Tibet, China | MK991804 | MN013772 | MN013778 |
| *Goodyera hachijoensis* | 46 (HSNU) | Chung | Cibodas, Indonesia | _ | KT385521 | KT385608 |
| *Goodyera hemsleyana* | b14 (HSNU) | Hu | Malipo, Yunnan, China | KT344014 | KT385522 | KT385609 |
| *Goodyera henryi* | a10 (HSNU) | Tian | Cibagou, Tibet, China | KT344017 | KT385523 | KT385610 |
| *Goodyera henryi* | b32 (HSNU) | Hu | Wenchuan, Sichuan, China | KT344018 | KT385524 | KT385611 |
| *Goodyera henryi* | b36 (HSNU) | Hu & Li | Emeishan, Sichuan, China | KT344019 | KT385525 | KT385612 |
| *Goodyera hispida* | b86 (HSNU) | Tian | Modog, Tibet, China | KT344020 | KT385526 | KT385613 |
| *Goodyera hispida* | c12 (HSNU) | Jiang | _ | KT344021 | KT3855227 | KT385614 |
| *Goodyera kwangtungensis* | b16 (HSNU) | Hu | Malipo, Yunnan, China | KT344025 | KT385529 | KT385616 |
| *Goodyera kwangtungensis* | b30 (HSNU) | Hu & Li | Nanchuan, Chongqing, China | KT344027 | KT385531 | KT385618 |
| *Goodyera kwangtungensis* | b44 (HSNU) | Hu | Mao’ershan, Guangxi, China | KT344029 | KT385533 | KT385620 |
| *Goodyera malipoensis* | Chen et al. Liu Z.J. 7997 (FAFU, NOCC) | Chen et al. | Malipo, Yunnan, China | KM593691 | _ | _ |
| *Goodyera malipoensis* | Chen et al. Liu Z.J. 8071 | Chen et al. | Malipo, Yunnan, China | KM593692 | _ | _ |
| *Goodyera marginata* | 503 (HSNU) | Chung | Chengdu, Sichuan, China | KT344030 | KT385534 | KT385621 |
| *Goodyera marginata* | a13-4 (HSNU) | Tian | Bome, Tibet, China | KT344032 | KT385535 | KT385622 |
| *Goodyera marginata* | b06 (HSNU) | Hu | Dali, Yunnan, China | KT344033 | KT385536 | KT385623 |
| *Goodyera medogensis* | 21062209 (HSNU) | Huang & Sun | Medog, Tibet, China | OL333866 | OL312072 | OL312076 |
| *Goodyera medogensis* | 21062310 (HSNU) | Huang & Sun | Medog, Tibet, China | OL333867 | OL312073 | OL312077 |
| *Goodyera medogensis* | 21062312 (HSNU) | Huang & Sun | Medog, Tibet, China | OL333868 | OL312074 | OL312078 |
| *Goodyera medogensis* | 21062216 (HSNU) | Huang & Sun | Medog, Tibet, China | OL333869 | OL312075 | OL312079 |
| *Goodyera nankoensis* | b33 (HSNU) | Hu & Li | Emeishan, Sichuan, China | KT344036 | KT385537 | KT385624 |
| *Goodyera nankoensis* | b57 (HSNU) | Ge | Bome, Tibet, China | KT344037 | KT385538 | KT385625 |
| *Goodyera pendula* | 525 (HSNU) | Chung | Ilan, Taiwan, China | KT344039 | KT385539 | KT385626 |
| *Goodyera pendula* | 629 (HSNU) | Chung | Taitung, Taiwan, China | KT344040 | KT385540 | KT385627 |
| *Goodyera pendula* | a16 (HSNU) | Tian | Nanling, Guangdong, China | KT344041 | KT385541 | KT385628 |
| *Goodyera pendula* | e038 (HSNU) | Qin | Mao’ershan, Guangxi, China | MK991805 | MN013773 | MN032023 |
| *Goodyera prainii* | 707_2 (HSNU) | Tian & Hu | Yingjiang, Yunnan, China | KT344042 | KT385542 | KT385629 |
| *Goodyera procera* | b01 (HSNU) | Hu | South China Botanical Garden, Guangdong | KT344044 | KT385543 | KT385630 |
| *Goodyera procera* | b69 (HSNU) | Tian & Dong | Wuzhishan, Hainan, China | KT344045 | KT385544 | KT385631 |
| *Goodyera pusilla* | a20 (HSNU) | Tian | Ruyuan, Guangdong, China | KT344046 | KT385545 | KT385632 |
| *Goodyera pusilla* | b100 (HSNU) | Tian | Taitung, Taiwan, China | KT344047 | KT385546 | KT385633 |
| *Goodyera repens* | 632 (HSNU) | Chung | Deqin County, Yunnan, China | KT344048 | KT385547 | KT385634 |
| *Goodyera repens* | a13-1 (HSNU) | Tian | Bome, Tibet, China | KT344049 | KT385548 | KT385635 |
| *Goodyera repens* | b48 (HSNU) | Wei | Mt.Tianshan, Xinjiang, China | KT344050 | KT385549 | KT385636 |
| *Goodyera repens* | b49 (HSNU) | Wei | Mt.Tianshan, Xinjiang, China | KT344051 | KT385550 | KT385637 |
| *Goodyera robusta* | 541 (HSNU) | Chung | Mt. Fansipan, Sapa, Vietnam | KT344054 | KT385552 | KT385639 |
| *Goodyera rubicunda* | b88 (HSNU) | Tian | Bawangling, Hainan, China | KT344060 | KT385555 | KT385642 |
| *Goodyera rubicunda* | b101 (HSNU) | Tian | Taitung, Taiwan, China | KT344058 | KT385553 | KT385640 |
| *Goodyera rubicunda* | b104 (HSNU) | Tian | Hualien, Taiwan, China | KT344059 | KT385554 | KT385641 |
| *Goodyera schlechtendaliana* | a14 (HSNU) | Tian | Nyingchi, Tibet, China | KT344065 | KT385556 | KT385643 |
| *Goodyera schlechtendaliana* | b85 (HSNU) | Tian | Nyingchi, Tibet, China | KT344067 | KT385558 | KT385645 |
| *Goodyera schlechtendaliana* | b106 (HSNU) | Tian | Taoyuan, Taiwan, China | KT344066 | KT385557 | KT385644 |
| *Goodyera seikomontana* | 109 (HSNU) | Chung | Ilan, Taiwan, China | KT344068 | KT385560 | KT385647 |
| *Goodyera seikomontana* | a22 (HSNU) | Tian | Nanling, Guangdong, China | KT344069 | KT385559 | KT385646 |
| *Goodyera thailandica* | b77 (HSNU) | Ye | Simao, Yunnan, China | KT344070 | KT385561 | KT385648 |
| *Goodyera velutina* | b04 (HSNU) | Hu | Tianmu Mountain, Zhejiang, China | KT344073 | KT385562 | KT385649 |
| *Goodyera velutina* | b31 (HSNU) | Hu & Li | Sanjiang Ecological Tourist Park, Sichuan, China | KT344075 | KT385564 | KT385651 |
| *Goodyera velutina* | b105 (HSNU) | Tian | Taoyuan County, Taiwan, China | KT344074 | KT385563 | KT385650 |
| *Goodyera viridiflora* | 524 (HSNU) | Chung | Hsinchu, Taiwan, China | KT344080 | KT385567 | KT385654 |
| *Goodyera viridiflora* | a19 (HSNU) | Tian | Nanling, Guangdong, China | KT344082 | KT385568 | KT385655 |
| *Goodyera viridiflora* | b19 (HSNU) | Hu | Malipo, Yunnan, China | KT344084 | KT385570 | KT385657 |
| *Goodyera viridiflora* | b107 (HSNU) | Chung | Philippines | KT344083 | KT385569 | KT385656 |
| *Goodyera vittata* | 706 (HSNU) | Tian & Hu | Yingjiang, Yunnan, China | KT344086 | KT385572 | KT385659 |
| *Goodyera wolongensis* | c13 (HSNU) | Tang | Wanglang, Sichuan, China | KT344087 | KT385573 | KT385660 |
| *Goodyera wolongensis* | e020-1 (HSNU) | Tian, Zhang & Zhou | Songpan, Sichuan, China | MK991806 | MN013779 | MN013774 |
| *Goodyera wolongensis* | e021-4 (HSNU) | Tian, Zhang & Zhou | Pingwu, Sichuan, China | MK991807 | MN013780 | MN013775 |
| *Goodyera yamiana* | 78 (HSNU) | Chung | Taitung, Taiwan, China | KT344088 | KT385574 | KT385661 |
| *Goodyera yunnanensis* | a13-2 (HSNU) | Tian | Bome, Tibet, China | KT344089 | KT385575 | KT385662 |
| *Goodyera yunnanensis* | a13-3 (HSNU) | Tian | Bome, Tibet, China | KT344090 | KT385576 | KT385663 |
| *Zeuxine flava* | b028 (HSNU) | Hu | Yingjiang, Yunnan, China | MT872173 | MT872618 | MT887724 |

Note: “_” means missing data.

Table. S2 Primers for sequence amplification and sequencing.

| Sequence | Primers（5’-3’） | Reference | Amplication protocol |
| --- | --- | --- | --- |
| ITS  *trn*L-F | 18S dir: CGTAACAAGGTTTCCGTAGG | Venora et al., 2000 ^[1]^ | 92°C 3min; 95°C 30s, 50°C 30s, 72°C 1min, 30cycles; 72°C 7min  94°C 2min; 94°C 1min, 50°C 30s, 72°C 1min, 30cycles; 72°C 7min |
|  | ITS4: TCCTCCGCTTATTGATATGC | White et al., 1990 ^[2]^ |  |
|  | c: CGAAATCGGTAGACGCTACG  f: ATTTGAACTGGTGACACGAG | Taberlet et al., 1991 ^[3]^  Taberlet et al., 1991 ^[3]^ |  |
| *mat*K 1  *mat*K 2 | 19F: CGTTCTGACCATATTGCACTATG  834R: AAAGACTCCARAAGATRTTG | Molvray et al., 2000 ^[4]^  Kocyan et al., 2004 ^[5]^ | 80°C 5min; 95°C 1min, 51°C 1min, 65°C 4min, 30cycles; 65°C 5min  The same as *mat*K 1 |
|  | 731F: TCTGGAGTCTTTCTTGAGCGA  trnK2R: AACTAGTCGGATGGAGTAG | Gervendeel et al., 2001 ^[6]^  Johnson & Soltis, 1994 ^[7]^ |  |

1. Venora G, Blangiforti S, Frediani M, Maggini E, Gelati MT. Nulclear DNA contents, rDNAs, chromatin organization, and karyotype evolution in *Vicia* sect. *faba*. Protoplasma. 2000; 213: 118-125.
2. White TJ, Bruns T, Lee S, Taylor JW. Ampliﬁcation and direct sequencing of fungal ribosomal RNA genes for phylogenetics, In Innis MA, Gelfand DH, Sninsky JJ, White TJ. PCR Protocols: A guide to methods and applications, San Diego: Academic Press; 1990, pp. 315-322.
3. Taberlet P, Gielly L, Pautou G, Bouvet J. Universal primers for amplification of three non-coding regions of chloroplast DNA. Plant Mol Biol. 1991; 17: 1105-1110.
4. Molvray M, Kores PJ, Chase MW. Polyphyly of mycoheterotrophic orchids and functional influences on floral and molecular characters. In: Wilson KL, Morrison DA, Monocots: Systematics and Evolution. Collingwood: CSIRO; 2000. pp. 441-448.
5. Kocyan A, Qiu YL, Endress PK, Conti EA. Phylogenetic analysis of Apostasioideae (Orchidaceae) based on ITS, *trnL-F* and *matK* sequences. Plant Syst Evol. 2004; 247: 203-213.
6. Gravendeel B, Chase MW, Vogel de EF, [Roos](https://sslvpn.ntu.edu.tw/,DanaInfo=www.amjbot.org+search?author1=Marco+C.+Roos&sortspec=date&submit=Submit) MC, [Mes](https://sslvpn.ntu.edu.tw/,DanaInfo=www.amjbot.org+search?author1=Ted+H.+M.+Mes&sortspec=date&submit=Submit), THM, Bachmann K. Molecular phylogeny of *Coelogyne* (Epidendroideae; Orchidaceae) based on plastid RFLPS, *matK*, and nuclear ribosomal ITS sequences: evidence for polyphyly. Amer J Bot. 2001; 88(10): 1915-1927.
7. Johnson LA, Soltis DE. *MatK* DNA sequences and phylogenetic reconstruction in Saxifragranceae s. str. Syst Bot. 1994; 19: 143-156.
